# Draft Genome Sequences of the Probiotic *Enterococcus faecalis* Symbioflor 1 Clones DSM16430 and DSM16434

**DOI:** 10.1128/genomeA.01061-16

**Published:** 2016-09-29

**Authors:** Moritz Fritzenwanker, Anindita Chakraborty, Torsten Hain, Kurt Zimmermann, Eugen Domann

**Affiliations:** a Institute of Medical Microbiology, German Centre for Infection Research (DZIF Partner Site Giessen-Marburg-Langen), Justus-Liebig-University Giessen, Giessen, Germany; b SymbioGruppe GmbH & Co KG, Herborn, Germany

## Abstract

The probiotic Symbioflor 1 is a historical concoction of 10 isolates of *Enterococcus faecalis*. Pulsed-field gel electrophoresis revealed two groups: one comprising eight identical clones (DSM16430, DSM16432, DSM16433, DSM16435 to DSM16439) and a further two isolates (DSM16431, DSM16434) with marginally different profiles. Here, we report a comparative analysis of the draft genome sequences of representative isolates.

## GENOME ANNOUNCEMENT

The homeostasis of gut microbiota is essential for the maintenance of health, and infectious or metabolic imbalance can lead to gastrointestinal disorders (1–3). Modulation of the gut microbiota to prevent disease is therefore an area of great interest. The conceptual use of beneficial probiotic bacteria in regulating intestinal dysbiosis has been propagated, and the use of these microorganisms has been implicated in promoting health following consumption (4, 5).

The commercially available probiotic Symbioflor 1 (SymbioPharm, Herborn, Germany) comprises 10 probiotic *Enterococcus faecalis* isolates obtained from a healthy volunteer in the early 1950s. *Enterococci* spp. are Gram-positive, facultative anaerobic cocci that reside as commensals in the oral cavity, vagina, and gastrointestinal tract of humans. These bacteria can cause a broad range of diseases, including urinary tract infections, endocarditis, and peritonitis (6). However, species of the genus *Enterococcus* have gained great interest due to their dual-faced status of being a common commensal in the human microbiota as well as displaying pathogenic traits (7). *Enterococci* spp. have been used as starters or probiotic cultures that are commonly found in traditional Mediterranean food and fermented food (4, 8). Symbioflor 1 has been prescribed since 1954 in Germany as a pharmaceutical preparation intended to improve the microbial balance of the intestinal flora with no records of pathogenic incidents (9). Analytical pulsed-field gel electrophoresis (restriction enzyme SmaI fingerprinting) of the 10 isolates (DSM16430 to DSM16439) revealed eight identical clones (DSM16430, DSM16432, DSM16433, DSM16435 to DSM16439) and a further two isolates (DSM16431, DSM16434) exhibiting marginal differences. The complete genome sequence of one of these isolates, DSM16431, has been described previously (10). We determined the sequence of DSM16430 as a representative of the eight identical isolates together with DSM16434 and performed comparative genomics.

DNA sequencing libraries were prepared using the Nextera XT kit (Illumina, San Diego, CA, USA) according to the manufacturer’s instructions. Individually tagged libraries were sequenced as a part of a flow cell on the Illumina MiSeq platform with v3 chemistry (2 × 300 bp). Genomes were assembled with CLC Genomics Workbench and compared with Mauve (11).

For DSM16434 we generated 1,850,276 reads with an average length of 183 bp (gapped genome size: 2,763,070 bp). Mauve analysis (at least 90% identity and coverage) revealed ~99% identity with the genome of DSM16431 (10). The genome of DSM16430 was assembled to a gapped genome of 2,633,486 bp in size from a total of 2,270,870 reads with an average length of 179 bp. Comparative genome analysis indicated that the three genomes are highly homologous, sharing a core genome of ~88% (2,468 open reading frames). The genome size of DSM16430 is smallest and lacks two regions present in the other isolates. This includes a region of ~100 kb in size lacking multiple transposases as well as the aggregation substance; the smaller gap had a size of ~36 kb, and represented the loss of a region encoding for a prophage.

Our data indicate a clonal origin for the 10 *E. faecalis* isolates found in Symbioflor 1 and suggests that this probiotic preparation could effectively be reduced to a combination of only two clonal strains, DSM16430 and DSM16431.

### Accession number(s).

These whole-genome shotgun projects have been deposited in the European Nucleotide Archive under the accession numbers FLUS00000000 (DSM16430) and FLUT00000000 (DSM16434). The versions described in this paper are the first versions, FLUS01000000 and FLUT01000000.
